# Supplementary material for: Integrated single cell multiomic profiling and functional validation reveal distinct cellular routes to human plasma cell differentiation
Source: bioRxiv. 2026 Feb 18:2026.02.16.706247. Preprint. [Version 1] doi: 10.64898/2026.02.16.706247 (PMC12934664; doi:10.64898/2026.02.16.706247)
Supplement: Supplement 1 [file NIHPP2026.02.16.706247v1-supplement-1.pdf]

bioRxiv preprint doi: https://doi.org/10.64898/2026.02.16.706247; this version posted February 18, 2026. The copyright holder for this preprint (which was not certified by peer review) is the author/funder, who has granted bioRxiv a license to display the preprint in perpetuity. It is made available under aCC-BY 4.0 International license.

Supplemental Figure 1

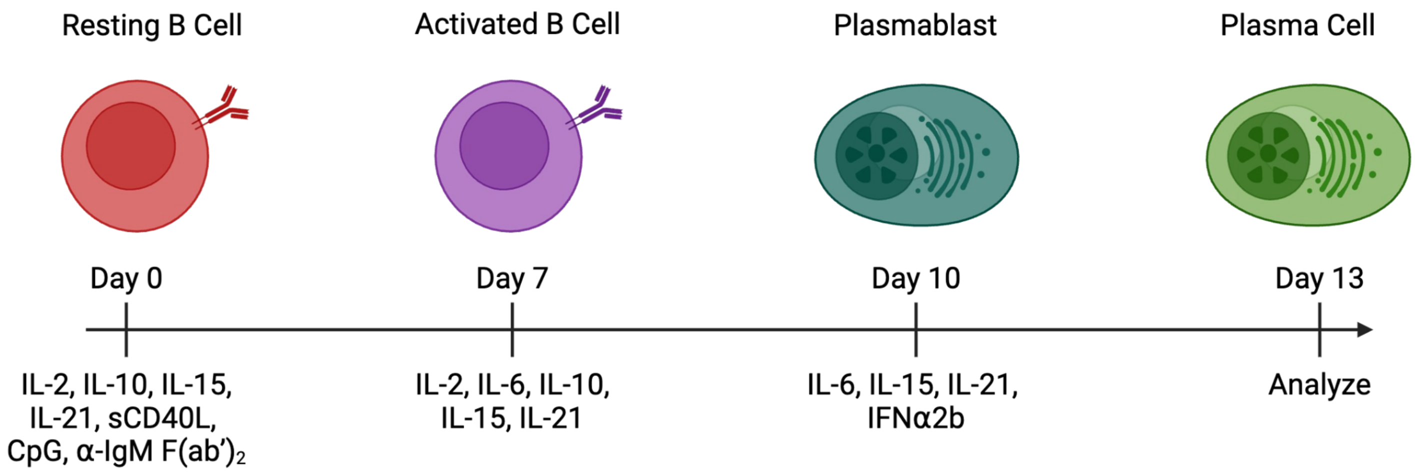

bioRxiv preprint doi: https://doi.org/10.64898/2026.02.16.706247; this version posted February 18, 2026. The copyright holder for this preprint (which was not certified by peer review) is the author/funder, who has granted bioRxiv a license to display the preprint in perpetuity. It is made available under aCC-BY 4.0 International license.

Sup. Fig. 1: *In vitro* differentiation scheme.

Primary tonsil naïve B cells were cultured with the indicated cytokines and mitogens to induce plasma cell differentiation.

bioRxiv preprint doi: https://doi.org/10.64898/2026.02.16.706247; this version posted February 18, 2026. The copyright holder for this preprint (which was not certified by peer review) is the author/funder, who has granted bioRxiv a license to display the preprint in perpetuity. It is made available under aCC-BY 4.0 International license.

Supplemental Figure 2

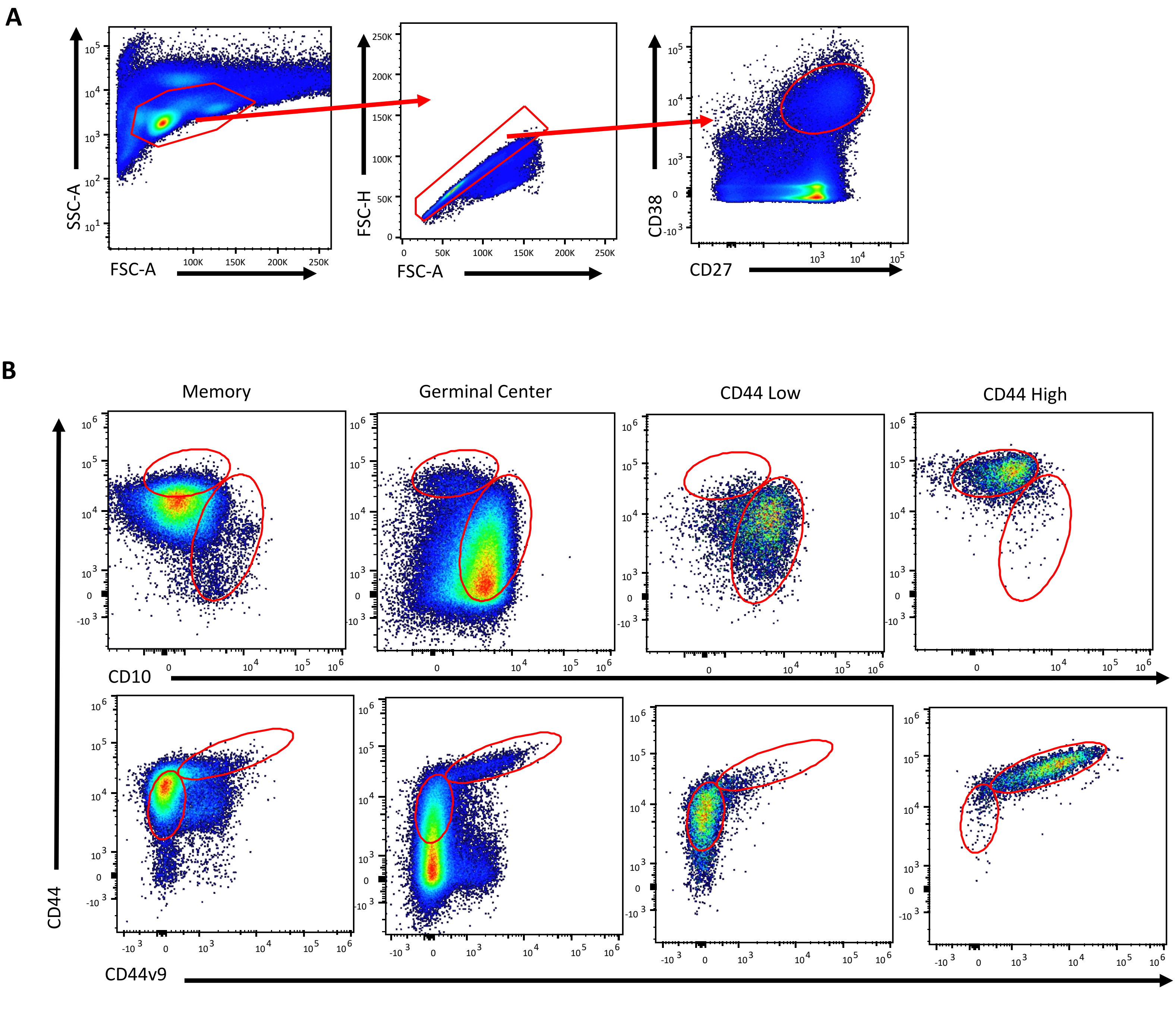

bioRxiv preprint doi: https://doi.org/10.64898/2026.02.16.706247; this version posted February 18, 2026. The copyright holder for this preprint (which was not certified by peer review) is the author/funder, who has granted bioRxiv a license to display the preprint in perpetuity. It is made available under aCC-BY 4.0 International license.

Sup. Fig. 2 Gating strategies for identification and comparison of tonsil plasma cell subsets.

(A) Gating scheme for bone marrow plasma cells (CD27+CD38+). **(B)** Comparison of CD44 and CD44v9 expression between tonsil plasma cell subsets and memory and germinal center B cells (n = 1 tonsil donor).

bioRxiv preprint doi: https://doi.org/10.64898/2026.02.16.706247; this version posted February 18, 2026. The copyright holder for this preprint (which was not certified by peer review) is the author/funder, who has granted bioRxiv a license to display the preprint in perpetuity. It is made available under aCC-BY 4.0 International license.

Supplemental Figure 3

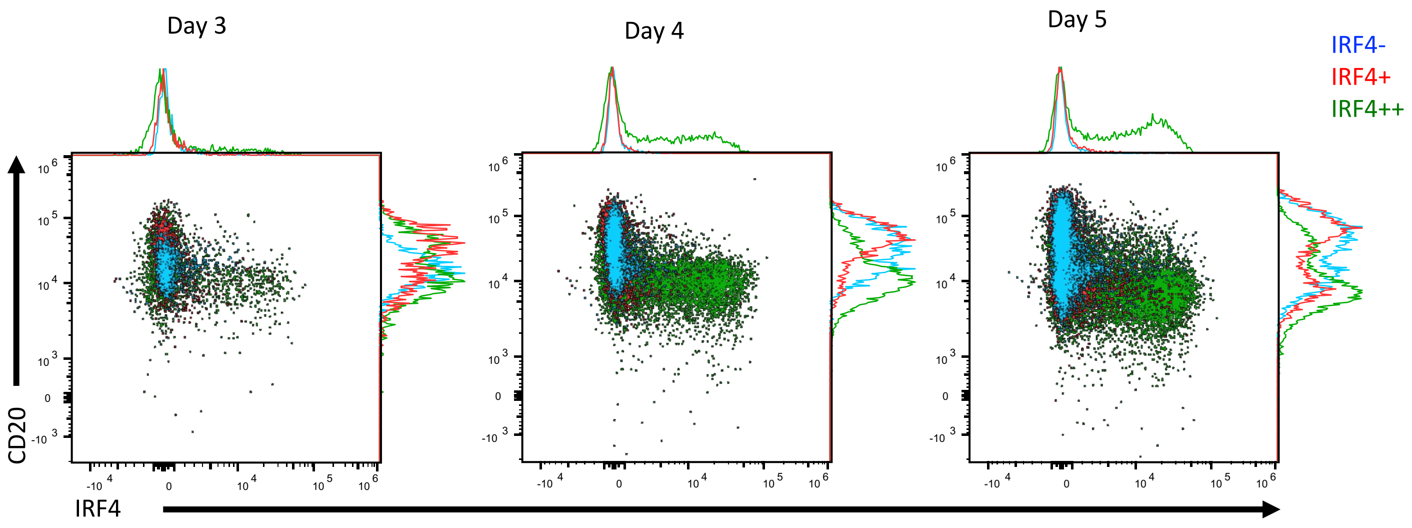

bioRxiv preprint doi: https://doi.org/10.64898/2026.02.16.706247; this version posted February 18, 2026. The copyright holder for this preprint (which was not certified by peer review) is the author/funder, who has granted bioRxiv a license to display the preprint in perpetuity. It is made available under aCC-BY 4.0 International license.

Sup. Fig. 3: Changes in IRF4 and CD20 observed during *in vitro* differentiation.

(A) Comparison of CD20 expression in cells with varying levels of IRF4 expression during Day 3, 5, and 5 of *in vitro* differentiation staring from primary naïve B cells.

bioRxiv preprint doi: https://doi.org/10.64898/2026.02.16.706247; this version posted February 18, 2026. The copyright holder for this preprint (which was not certified by peer review) is the author/funder, who has granted bioRxiv a license to display the preprint in perpetuity. It is made available under aCC-BY 4.0 International license.

Supplemental Figure 4

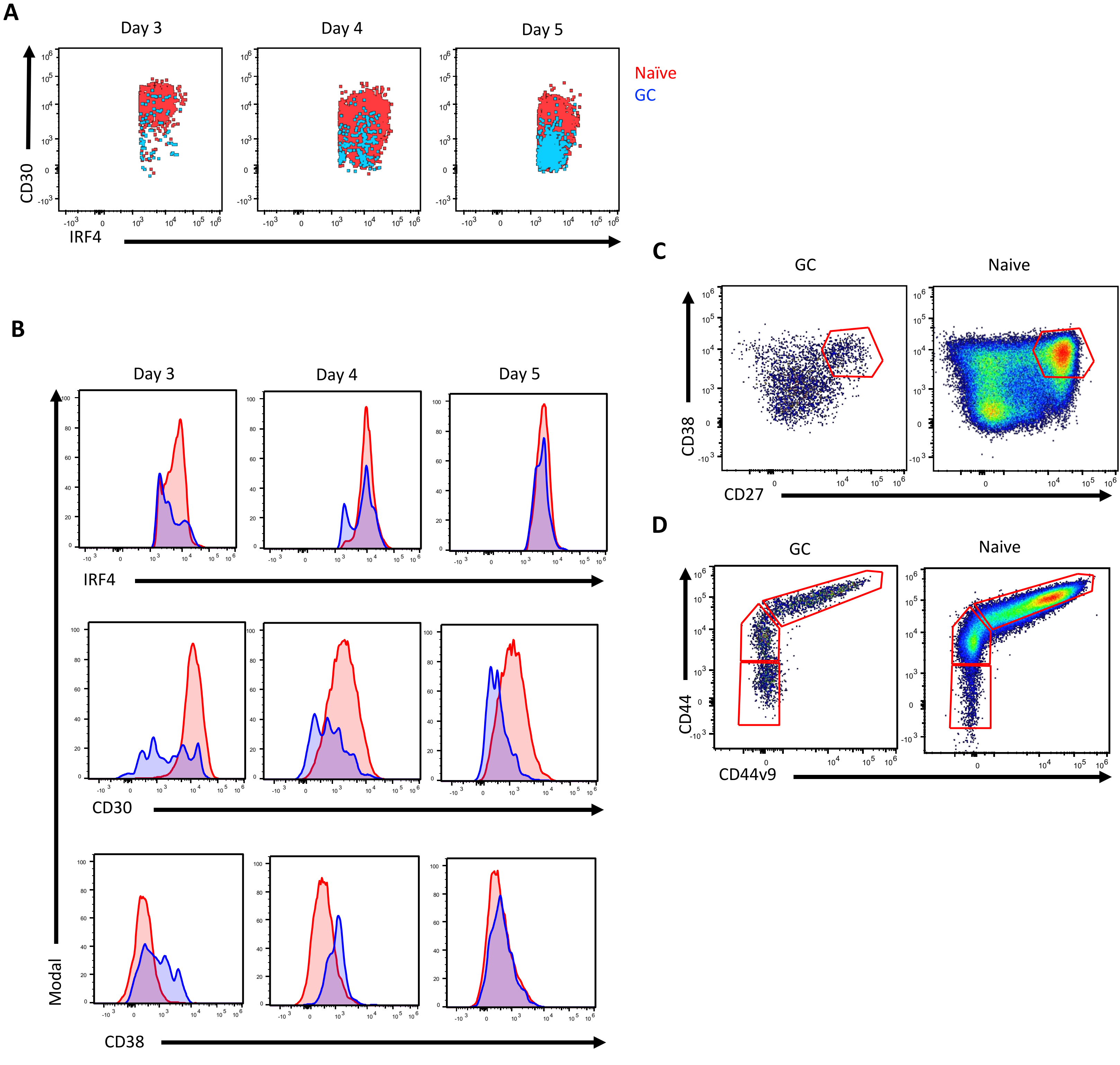

bioRxiv preprint doi: https://doi.org/10.64898/2026.02.16.706247; this version posted February 18, 2026. The copyright holder for this preprint (which was not certified by peer review) is the author/funder, who has granted bioRxiv a license to display the preprint in perpetuity. It is made available under aCC-BY 4.0 International license.

Sup. Fig. 4: Primary germinal center B cells fail to form the CD30- and IRF4-based populations observed with naïve B cell.

(A) Daily time course of CD30 and IRF4 expression for *in vitro* differentiating sorted naïve B cells or germinal center B cells showing only those with IRF4 expression above the level of unresponsive cells (best visualized in Fig 4B day 3). **(B)** Overlaid histograms for the highest IRF4 expressing cells looking at IRF4, CD30, and CD38 expression. **(C)** Representative CD27 and CD38 profiles at the end of differentiation starting from germinal center or naïve B cells. **(D)** Representative CD44 and CD44v9 profiles at the end of differentiation starting from germinal center or naïve B cells.

bioRxiv preprint doi: https://doi.org/10.64898/2026.02.16.706247; this version posted February 18, 2026. The copyright holder for this preprint (which was not certified by peer review) is the author/funder, who has granted bioRxiv a license to display the preprint in perpetuity. It is made available under aCC-BY 4.0 International license.

Supplemental Figure 5

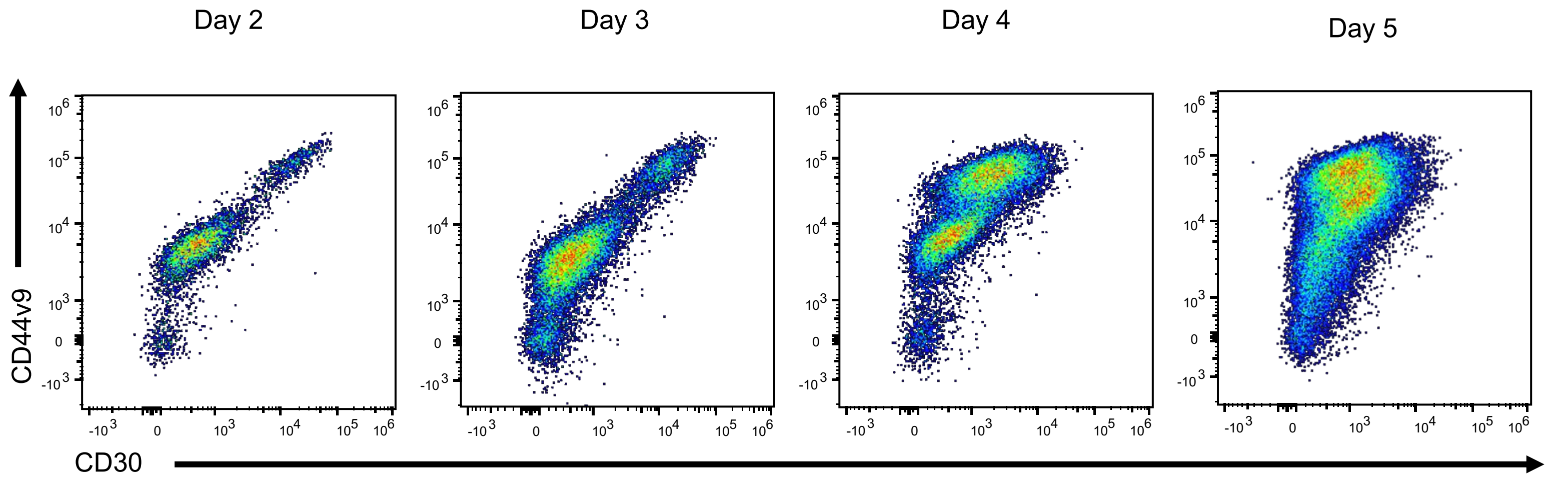

bioRxiv preprint doi: https://doi.org/10.64898/2026.02.16.706247; this version posted February 18, 2026. The copyright holder for this preprint (which was not certified by peer review) is the author/funder, who has granted bioRxiv a license to display the preprint in perpetuity. It is made available under aCC-BY 4.0 International license.

Sup. Fig. 5: Validation of linked CD30 and CD44v9 expression.

Daily time course of CD30 and CD44v9 expression of *in vitro* differentiating naïve B cells.

bioRxiv preprint doi: https://doi.org/10.64898/2026.02.16.706247; this version posted February 18, 2026. The copyright holder for this preprint (which was not certified by peer review) is the author/funder, who has granted bioRxiv a license to display the preprint in perpetuity. It is made available under aCC-BY 4.0 International license.

Supplemental Figure 6

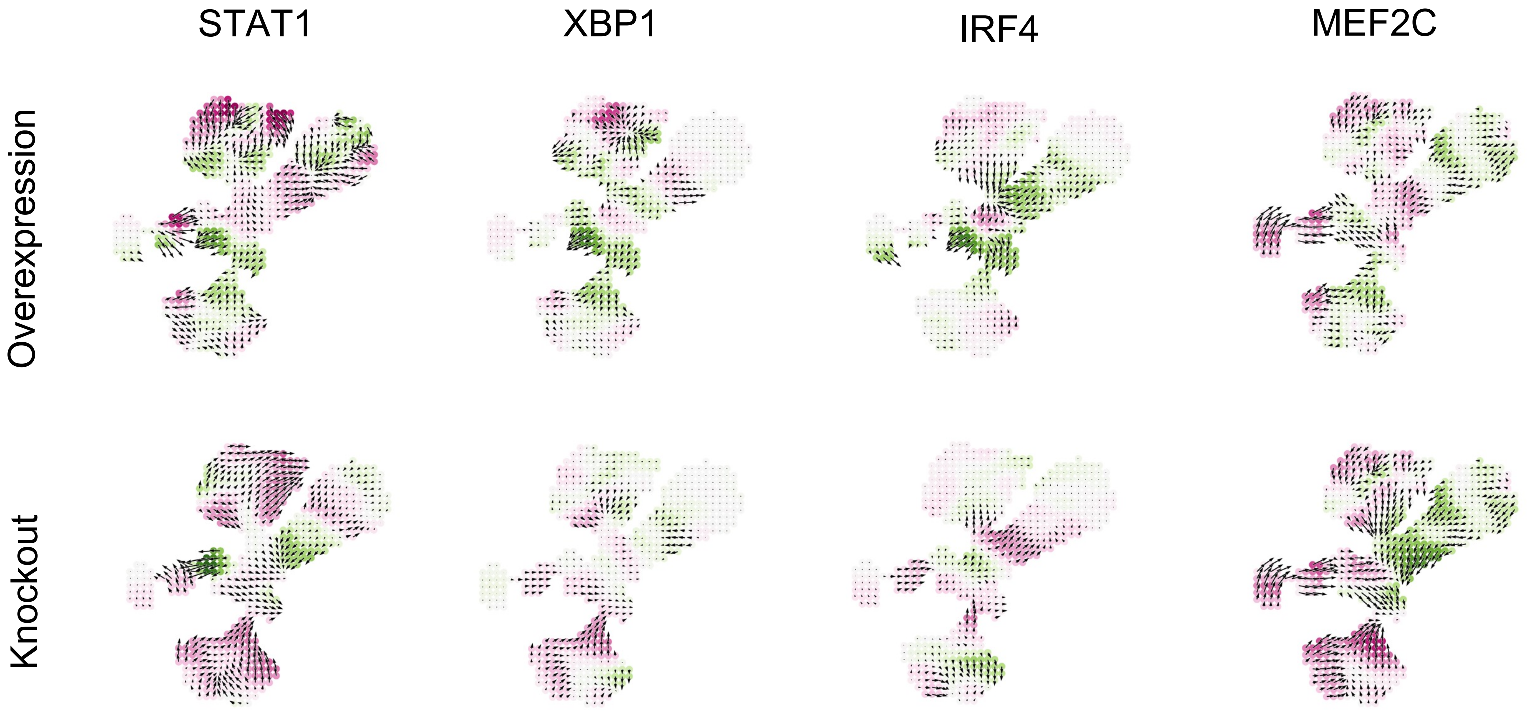

bioRxiv preprint doi: https://doi.org/10.64898/2026.02.16.706247; this version posted February 18, 2026. The copyright holder for this preprint (which was not certified by peer review) is the author/funder, who has granted bioRxiv a license to display the preprint in perpetuity. It is made available under aCC-BY 4.0 International license.

Sup. Fig. 6: Simulated overexpression or knockout of transcription factors identified by CellOracle analysis.

Overexpression was assessed by a simulated 10 to 20% increase in transcription factor activity and complete knockout for STAT1, XBP1 (as a surrogate for rosiglitazone activity), IRF4, and MEF2C. Green coloration indicates changes predicted to progress towards the plasma cell identity while purple coloration indicates predicted changes away from the plasma cell identity.

bioRxiv preprint doi: https://doi.org/10.64898/2026.02.16.706247; this version posted February 18, 2026. The copyright holder for this preprint (which was not certified by peer review) is the author/funder, who has granted bioRxiv a license to display the preprint in perpetuity. It is made available under aCC-BY 4.0 International license.

Supplemental Figure 7

A

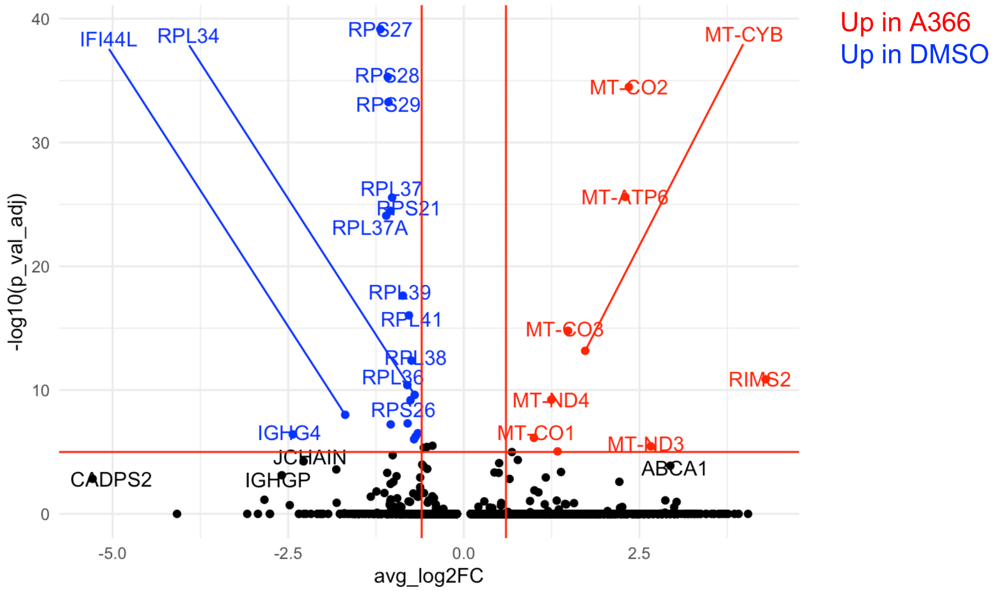

B

Gene	Log2FC	Adjusted p-value
MT-ND2	-0.3550	0.0000
MT-ND3	0.1370	0.0246
MT-ND4	-0.2430	0.0153
MT-CYB	-0.2231	0.0005
MT-CO1	-0.1792	0.0087
MT-CO2	-0.2069	0.0016
MT-CO3	-0.1714	0.0048
MT-ATP6	-0.2012	0.0110
RIMS2	-0.3555	0.0174
RPS27	0.2366	0.0656
RPL34	0.2686	0.0118
RPL36	#N/A	#N/A
RPL37	0.1935	0.0070
RPL37A	0.3010	0.0019
RPL38	0.2714	0.0122
RPL39	0.3052	0.0184
RPL41	0.1879	0.0085
RPS21	0.1547	0.0868
RPS26	0.1741	0.0579
RPS28	#N/A	#N/A
RPS29	0.3188	0.0121
IFI44L	#N/A	#N/A

bioRxiv preprint doi: https://doi.org/10.64898/2026.02.16.706247; this version posted February 18, 2026. The copyright holder for this preprint (which was not certified by peer review) is the author/funder, who has granted bioRxiv a license to display the preprint in perpetuity. It is made available under aCC-BY 4.0 International license.

Sup. Fig. 7: Differentially expressed genes in A366 treated cells match with MEF2C direct targets.

(A) Differentially expressed genes between A366 and DMSO treated cells. **(B)** Log-2-fold change and adjusted p-values for the genes identified in **(A)** comparing MEF2C knockout cells to wildtype cells reported in Ref. (Ow et al., 2016).

bioRxiv preprint doi: https://doi.org/10.64898/2026.02.16.706247; this version posted February 18, 2026. The copyright holder for this preprint (which was not certified by peer review) is the author/funder, who has granted bioRxiv a license to display the preprint in perpetuity. It is made available under aCC-BY 4.0 International license.

Supplemental Figure 8

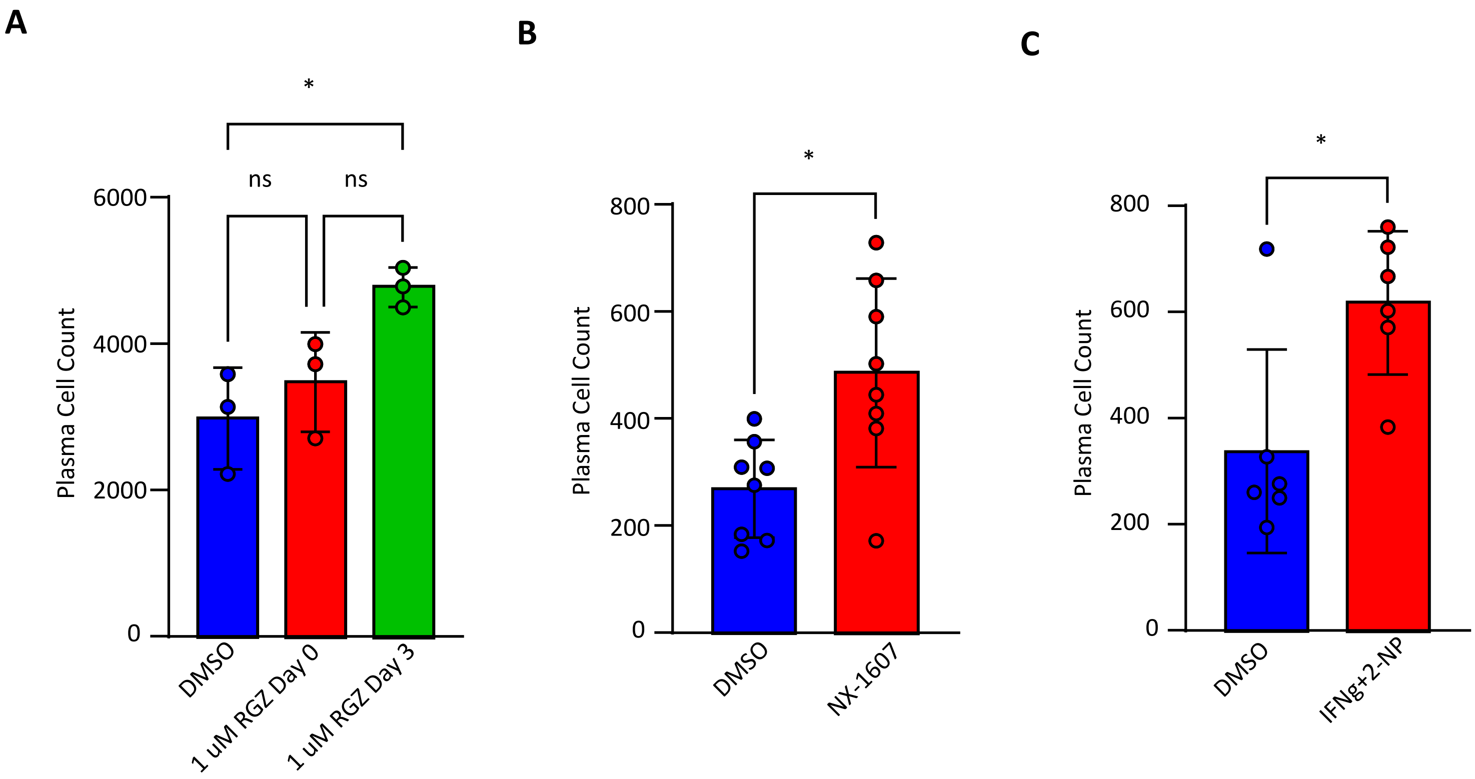

bioRxiv preprint doi: https://doi.org/10.64898/2026.02.16.706247; this version posted February 18, 2026. The copyright holder for this preprint (which was not certified by peer review) is the author/funder, who has granted bioRxiv a license to display the preprint in perpetuity. It is made available under aCC-BY 4.0 International license.

Sup. Fig. 8: Increased Xbp1, IRF4, and STAT signaling improve plasma cell yields.

Final plasma cell yields for **(A)** Rosiglitazone added on Day 0 or 3, **(B)** NX-1607 added on Day 5, or **(C)** Interferon-γ and 2-NP added on Day 5. Statistical significance was evaluated using a t-test in figures where only two groups are being compared. In all other instances, one-way analysis of variance (ANOVA) was performed to test for statistical significance. Data are presented as mean ± standard error of the mean (SEM). Statistical significance was defined as $*p < 0.05$.

bioRxiv preprint doi: https://doi.org/10.64898/2026.02.16.706247; this version posted February 18, 2026. The copyright holder for this preprint (which was not certified by peer review) is the author/funder, who has granted bioRxiv a license to display the preprint in perpetuity. It is made available under aCC-BY 4.0 International license.

Supplemental Figure 9

A

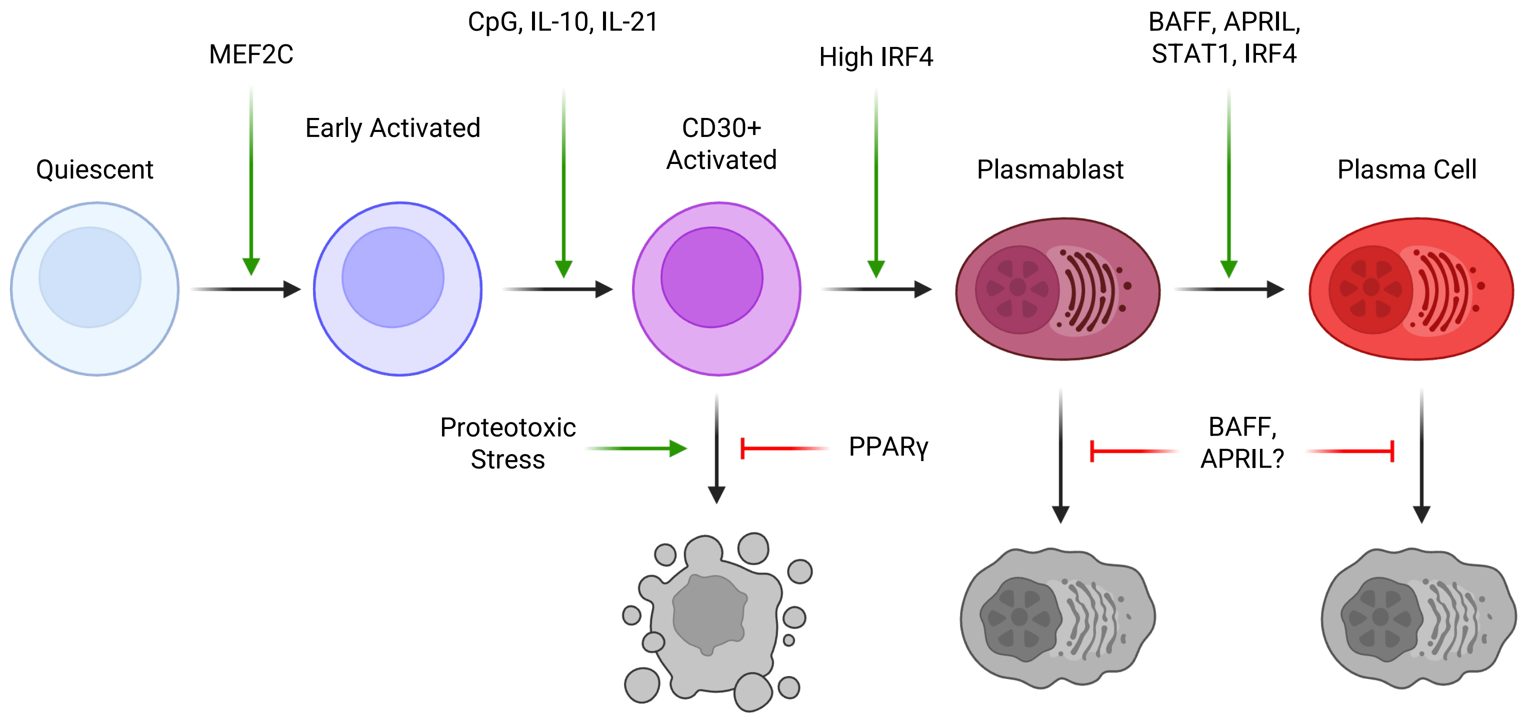

B

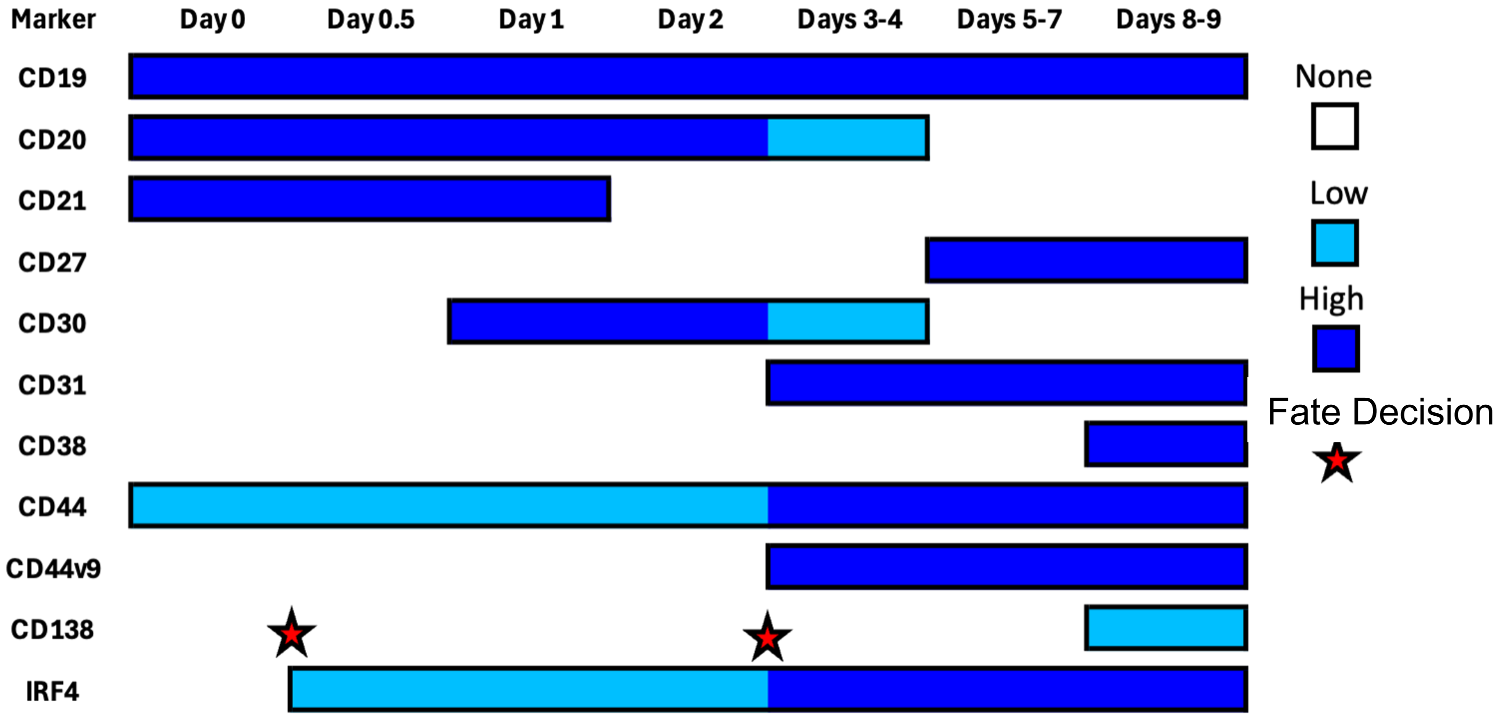

bioRxiv preprint doi: https://doi.org/10.64898/2026.02.16.706247; this version posted February 18, 2026. The copyright holder for this preprint (which was not certified by peer review) is the author/funder, who has granted bioRxiv a license to display the preprint in perpetuity. It is made available under aCC-BY 4.0 International license.

Sup. Fig. 9: Regulation of plasma cell differentiation and characteristic changes observed during differentiation.

(A) Identified and functionally validated regulators of different stages of plasma cell differentiation. **(B)** Changes in protein expression observed during *in vitro* plasma cell differentiation. Potential fate decisions are indicated with a red star.
